# Oncological and functional outcomes following transoral laser microsurgery in patients with T2a vs T2b glottic squamous cell carcinoma

**DOI:** 10.1186/s40463-019-0346-7

**Published:** 2019-06-07

**Authors:** David Forner, Matthew H. Rigby, Robert D. Hart, Jonathan R. Trites, S. Mark Taylor

**Affiliations:** Division of Otolaryngology – Head & Neck Surgery, Department of Surgery, Dalhousie University, 5820 University Ave., 3rd Floor Dickson Bldg, Halifax, Nova Scotia B3H 2Y9 Canada

**Keywords:** Glottic cancer, Transoral laser microsurgery, T2 glottic, Functional outcomes, Vocal cord mobility

## Abstract

**Background:**

There is a paucity of evidence comparing oncological and voice outcomes between T2a and T2b glottic squamous cell carcinoma (SCC) patients treated with transoral laser microsurgery (TLM). This study identified functional and oncological outcomes in this cohort.

**Methods:**

Retrospective review of prospectively collected data of patients treated with TLM for T2 glottic SCC from 2003 to 2017.

**Results:**

In total, 75 patients were included. Five-year local control rates were significantly different between T2a and T2b patients (75.2% vs 57.0%, *p* = 0.022). There was no difference in five-year survival between patients with T2a disease and T2b disease (69.5% vs 73.4%, *p* = 0.627). There was no significant difference in mean VHI-10 scores in the pre-operative period (18.3 vs 21.4, *p* = 0.409). However, patients with T2b disease had significantly worse perceived voice outcomes post-operatively (6.6 vs 21.3 *p* = 0.001). Patients with T2a disease saw significant improvements in mean VHI-10 scores following surgery (18.3 vs 6.6, *p* = 0.000), while T2b patients did not (21.4 vs 21.3, *p* = 0.979). The overall laryngeal preservation rate was 94.7%, with 11.5% of T2b patients requiring salvage organ sacrifice.

**Conclusions:**

This study highlights positive functional outcomes for T2a glottic SCC. Patients with T2b disease appear to have significantly worse oncological and functional outcomes, including worse voice quality following surgery and higher rates of salvage laryngectomy.

## Background

Squamous cell carcinoma (SCC) is the most common form of laryngeal cancer. Of the three anatomical subsites of the larynx, glottic involvement is by far the most common, accounting for 60 to 75% of laryngeal cancers. Minimally invasive surgical techniques, such as transoral laser microsurgery (TLM), have allowed for glottic cancers to be treated with primary surgery [1].

The current American Joint Committee on Cancer (AJCC) staging system for glottic cancer does not substratify primary tumor involvement for T2 cancers. However, some authors have called for substratification by vocal cord mobility, with reduced vocal cord mobility (T2b) suggesting more significant tumor invasion [2]. Previous studies have demonstrated a detrimental effect on local control and patient survival in patients with reduced vocal cord mobility when undergoing treatment with radiotherapy [3].

Only a single study has compared oncological outcomes in patients treated with TLM between T2a and T2b [4]. Local control rates and patient survival in T2b tumors were found to be more similar to T3 tumors than T2a. Transoral laser microsurgery and radiotherapy offer satisfactory voice outcomes in early glottic cancers, with conflicting results in regards to superiority of specific modalities [5, 6]. No studies have examined the difference in functional voice outcomes between patients with T2a and T2b treated with TLM. We hypothesized that functional outcomes after TLM would be worse for those patients with T2b disease.

This study is the first to describe the effect of reduced vocal cord mobility on perceived voice outcomes following treatment by TLM in patients with T2 glottic SCC. Additionally, we offer further evidence for oncological outcomes in this patient group.

## Methods

### Patients, diagnosis, and functional outcomes

Retrospective chart review of prospectively collected data for all patients undergoing TLM resection of T2 glottic SCC at our institution was completed. All patients undergoing TLM surgery diagnosed with cT2 disease between January 2003 and July 2017 were included. Patients were excluded if the initial TLM procedure represented salvage surgery, and if they had previously received radiation treatment. Where indicated, patients were excluded when information was lacking from the chart review. Patients were excluded if vocal cord mobility was not adequately documented. Patients initially staged as cT2 were included regardless if they were upstaged to pT3. This inclusion facilitated an intention-to-treat process. Follow-up lengths were determined from time of initial surgical intervention. Patients were staged as T2 according to the appropriate American Joint Committee on Cancer (AJCC) edition at time of diagnosis, including AJCC 6th and AJCC 7th editions. There was no difference in the diagnosis of T2 between the two editions, and was defined as tumor extending to the supraglottis and/or subglottis, and/or with impaired vocal fold mobility. Patients were considered T2b when there was decreased vocal cord mobility during awake pharyngoscopy. Patients with normal vocal cord mobility were considered T2a.

Data was prospectively collected in a TLM database with Voice Handicap Index-10 questionnaire results. The Voice Handicap Index was originally developed as a 30 item questionnaire by Jacobson and colleagues to quantify the psychosocial consequences of voice disorders [7]. Rosen and colleagues then developed an abbreviated version of the VHI questionnaire, the VHI-10 [8]. Mean VHI-10 scores were compared between the pre-operative period and the one-year post-operative follow-up. Complications, including tracheotomy and requirement for gastrostomy tube (G-Tube) placement, were considered for the first post-operative year.

### Statistical analysis and ethics

Statistical analysis was completed using the commercially available software SPSS (v21; IBM, Chicago, Illinois). Categorical variables were compared using either Chi-square test or Fischer’s exact test. Continuous variables were compared using either Student’s T-Test or Mann-Whitney U-Test. Both patient survival and recurrence rates were compared using Kaplan-Meier curves, with significance determined by Mantel-Cox Log Rank test methods. Overall survival was calculated with events being considered any cause of patient death, with patients alive at time of last follow-up being censored. Local and locoregional recurrence rates were calculated with events being considered either local or regional recurrences, and patients with no previous recurrence at time of last follow-up, or at time of death, being censored. New primaries were not considered as locoregional recurrence for these calculations. Ultimate control with TLM alone was calculated using Kaplan-Meier analysis, with events being considered the need for non-TLM intervention (i.e., adjuvant chemotherapy or radiotherapy, or total laryngectomy). Therefore, patients with locoregional recurrence but requiring only repeat TLM were considered censored in these analyses.

Review of patient information and use of the institutional TLM database was approved by the research ethics board of the Nova Scotia Health Authority.

## Results

### Patient demographics and treatment details

In total, 75 patients were included in the study. Patient demographics are found in Table 1. Mean follow-up for T2a patients was 106 months (range 0 to 334 months) and 124 months (range 2 to 308 months) for T2b patients. Eight patients with T2a disease (17.4%) underwent adjuvant therapy, while one patient with T2b disease received adjuvant treatment (3.4%).

**Table 1:** Patient demographics and Lymph Node Involvement Staging

Variable	Number (% or range)	T2a	T2b
Total Patients	75 (100)	46 (61.3)	29 (38.6)
Male Gender	67 (89)	45 (98)	22 (76)
Mean Age (range)	66 years (34–87 years)	67 years (34–83 years)	63 years (46–87 years)
N staging			
N0	73 (96.3)	47 (96.0)	26 (100)
N1	1 (1.3)	1 (2.0)	0
N2a	1 (1.3)	1 (2.0)	0
N2b	0	0	0
N2c	0	0	0
N3	0	0	0

### Oncological outcomes

**Fig. 1:**
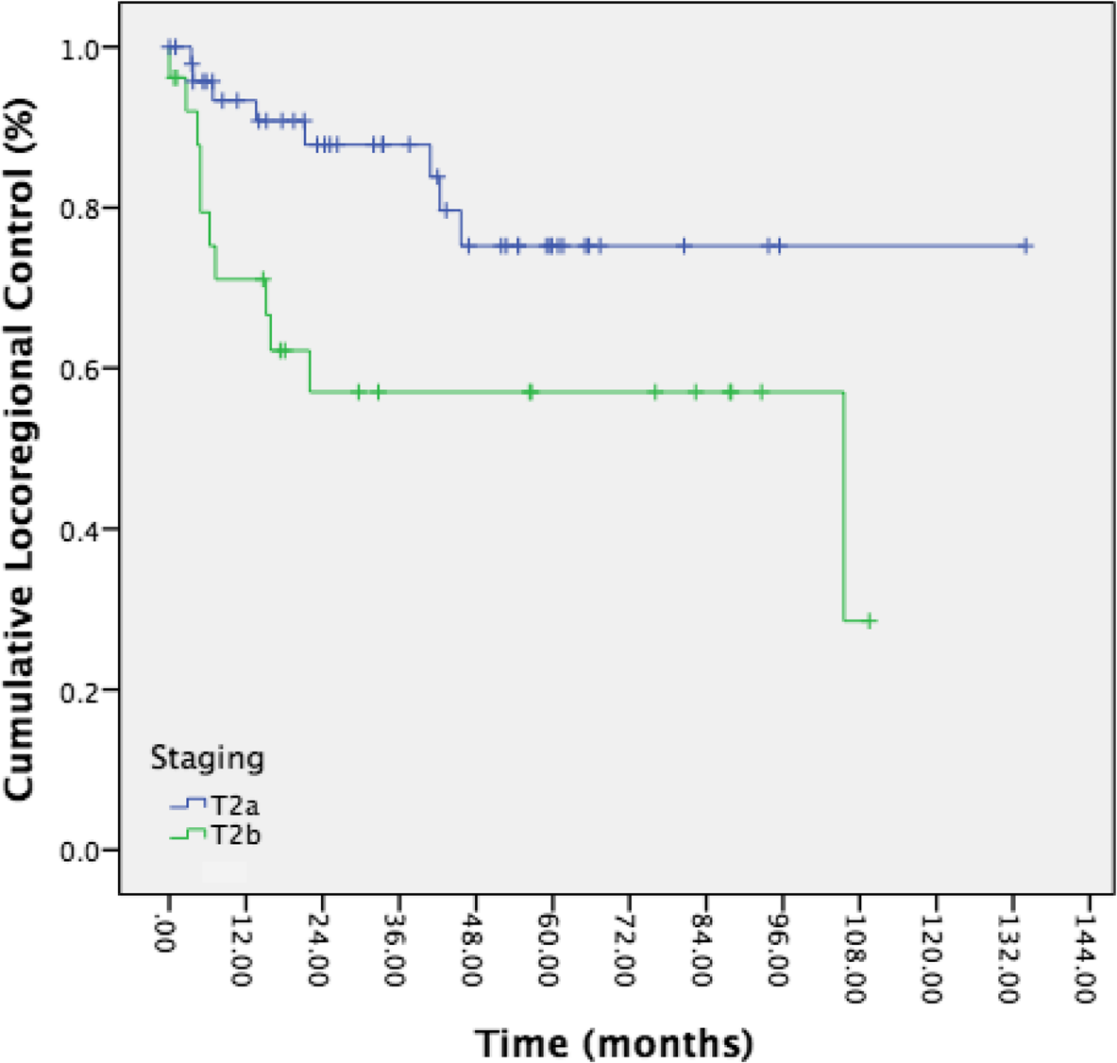
Kaplen-Meier curve representing recurrence rates of T2a (blue) vs T2b disease (green). Results were statistically significant by Log-Rank test statistic

In total, there were 19 local or locoregional recurrences. Five-year local and locoregional control was 69.1% when T2a and T2b were combined and analyzed as a single group. Five-year local and locoregional control rates after TLM were significantly different between T2a and T2b patients (Fig. 1, 75.2% vs 57.0%, *p* = 0.022). In this analysis, no patients with adjuvant therapy or repeat TLM were excluded. Eight patients with T2a disease developed local or locoregional recurrence (17.4%), compared to 11 patients in the T2b group (37.9%). Five-year ultimate local control with repeat TLM procedures was also significantly better for patients with T2a disease compared to those with T2b disease (87.9% vs 68.1%, *p* = 0.008). Three patients developed distant metastasis, one of which had T2b disease, and none of these patients had lymph node involvement on original presentation. Distant metastatic sites included lungs and liver.

**Fig. 2:**
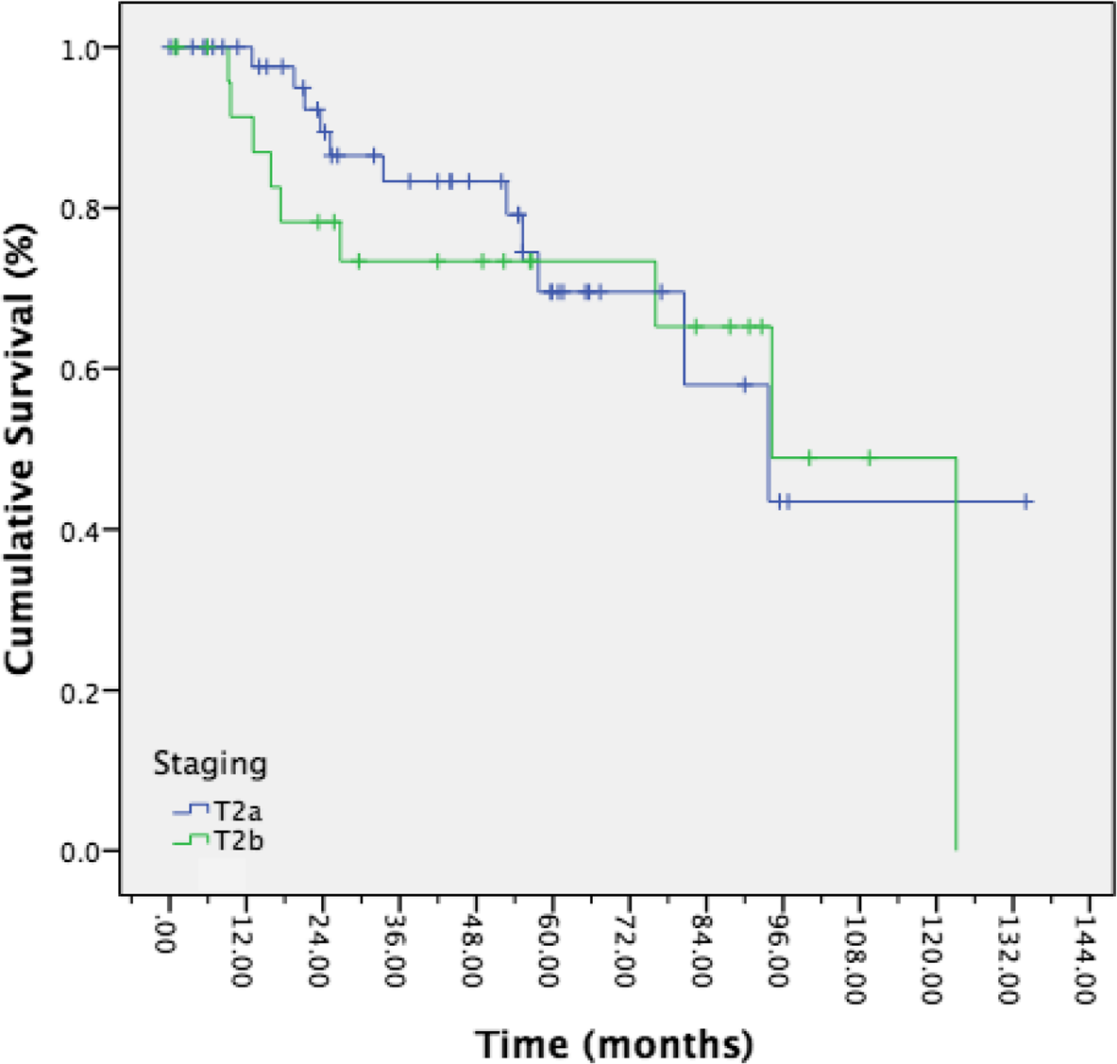
Kaplen-Meier curve representing overall survival of T2a (blue) vs T2b disease (green). Results were not statistically significant by Log-Rank test statistic

Twenty patients died over the course of the study. Seven patients died as a direct result of their disease. Overall five-year survival was 71.4%. There was no difference in five-year survival between patients with T2a disease and T2b disease (Fig. 2, 69.5% vs 73.4%, *p* = 0.627). Disease specific survival was calculated for T2b patients, as only one patient with T2a disease died due to disease. Of the T2b patients who died due to disease, none remained alive at 5 years. Mean survival time of these patients was 16 months. Notably, there was no difference in overall locoregional control or overall survival when patients with pre-operative clinical nodal disease were removed from the analyses.

### Functional outcomes

Functional complications within the first year of initial surgery were more common in patients with T2b disease. Two patients required tracheostomy tubes, both of which had T2b disease (7.6% vs 0%). One patient required a tracheostomy for management of a post-operative bleed following TLM. The other required a tracheostomy for relief of airway compromise in the setting of recurrent disease before 1 year. No patients required gastrostomy tube insertion.

In total, 58 patients had VHI-10 scores available for analysis. Voice Handicap Index-10 scores in the pre-operative period ranged from 1 to 38, and 0 to 28 post-operatively. Overall, there was a significant improvement in mean VHI-10 scores when the pre-operative and post-operative scores were compared (mean VHI-10 score 19.0 vs 9.0, *p* = 0.000). There was no significant difference in pre-operative VHI-10 score between T2a and T2b disease (mean VHI-10 score 18.3 vs 21.4, *p* = 0.409). However, there was a significant difference in post-operative VHI-10 score between the two groups (mean VHI-10 score 6.6 vs 21.3, p 0.001).

**Fig. 3:**
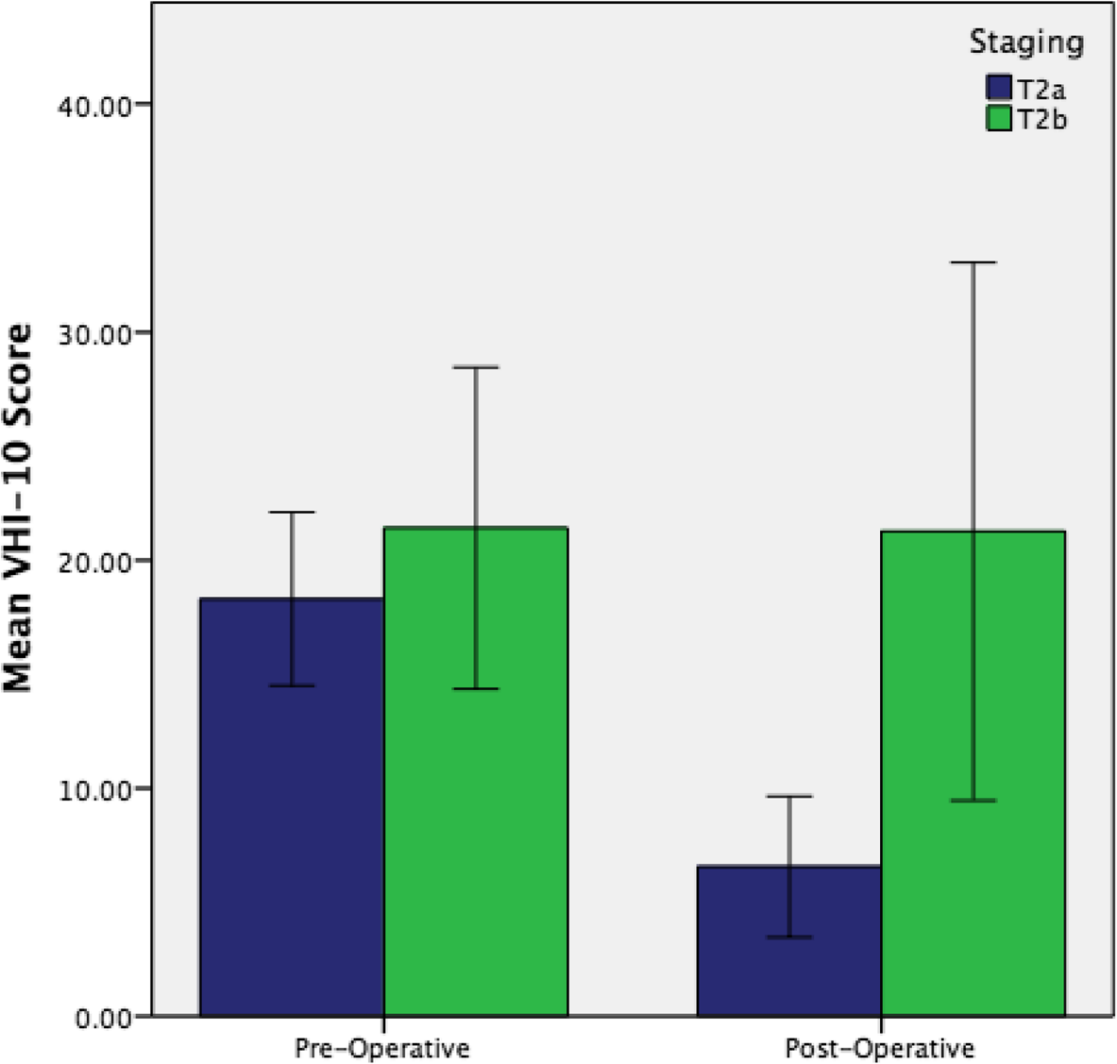
Pre-operative versus post-operative VHI-10 scores were significantly different for patients with T2a disease (blue) but not T2b disease (green). Errors bars represent 95% confidence intervals

Patients with T2a disease saw significant improvements in VHI-10 score following surgery (Fig. 3, mean VHI score 18.3 vs 6.6, *p* = 0.000). Patients with T2b disease did not experience significant improvement in voice quality following surgery (Fig. 3, mean VHI-10 21.4 vs 21.3, *p* = 0.979).

**Fig. 4:**
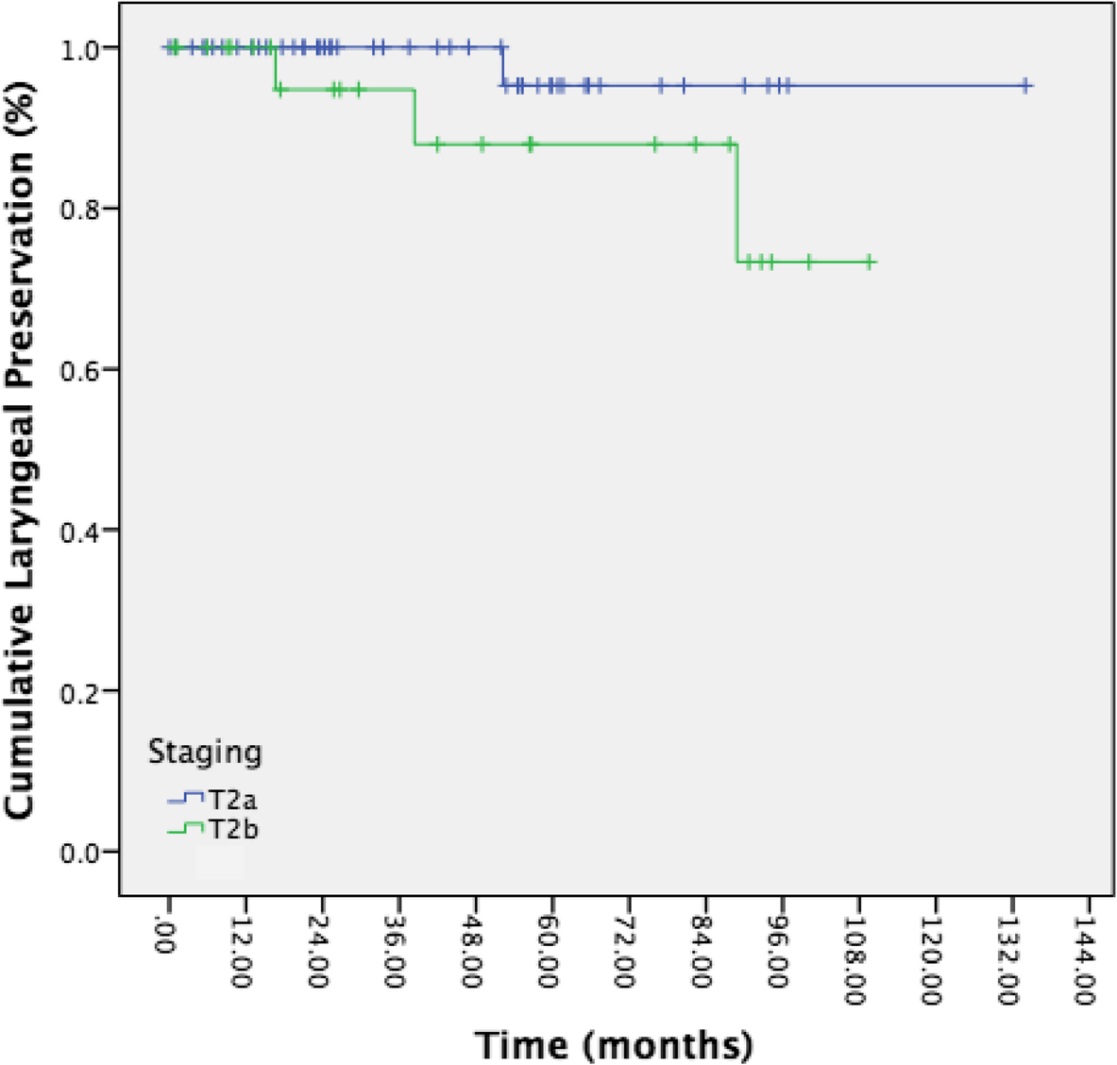
Kaplen-Meier curve representing laryngeal preservation rates of T2a (blue) vs T2b disease (green). Statistical significance was not tested due to low number of events in T2a

The overall laryngeal preservation rate was 94.7%, with all except one patient requiring organ sacrifice being T2b. In total, four total laryngectomies were required (11.5% of T2b patients). Patients with T2a disease had an organ preservation rate of 97.9%. Five-year laryngeal preservation rates non-significantly differed between patients with T2a disease and T2b disease (Fig. 4, 95.2% vs 88.0%, *p* = 0.140). One total laryngectomy in a patient with T2b disease was for functional purposes, in a patient with severe aspiration following multiple TLM surgeries as well as chemoradiation for repeated recurrences. The remainder of total laryngectomies were for advanced stage recurrences.

## Discussion

This is the first study to compare functional voice outcomes between T2a and T2b patients treated with transoral laser microsurgery, and only the second to identify differences in oncological outcomes between these groups. Reduced vocal cord mobility in T2 tumors signifies increased tumor invasion depth, and these tumors have traditionally behaved more similarly to T3 tumors, while T2a tumors are more closely related to T1 disease [9]. The question of substratification into T2a and T2b has therefore been raised in the past, as far back as 1967 [10], given the heterogeneous outcomes of T2 glottic cancer. The AJCC has not acknowledged a detriment in prognosis in patients with reduced vocal cord mobility.

Patients with T2b tumors treated with radiation therapy have been found to have worse locoregional control compared to T2a tumors. The large, randomized trial comparing hyperfractionation to conventional fractionation radiotherapy in T2 glottic cancer patients, RTOG 9512, demonstrated significantly worse local control, disease free survival, and overall survival in patients with T2b disease [11]. In a meta-analysis of retrospective studies by McCoul and Har-El, where the majority of studies examined radiotherapy alone, mean five-year local control rates for T2a cancers were 76.2%, compared to 64.4% for T2b cancers [3]. Patients with T2a disease in our population had similar results, while T2b disease had a slightly higher five-year recurrence rate. Our study also suggests that repeat TLM offers additional opportunity for locoregional control and is capable of sparing patients from adjuvant radiotherapy, perhaps preserving this adjuvant option for future treatment, if needed. This is especially true of T2a disease, with ultimate control with TLM alone reaching nearly 90%.

Canis and colleagues [4] showed a reduction in overall survival when vocal cord mobility was affected in T2 cancers. This finding was disputed in a meta-analysis by McCoul and Har-El. However, only five studies were included in the analysis of overall survival. There was no statistically significant difference in five-year survival demonstrated in our study. Despite this, oncological outcomes appear to be worse in terms of recurrence, necessitating further treatment. To further elucidate the impact of decreased vocal cord mobility on patient survival, a multi-center prospective trial is required.

Voice outcomes have not yet been compared between T2a and T2b patients treated with TLM [12]. Findings in this study show that T2b patients may not benefit from satisfactory patient perceived voice outcomes following TLM resection, with similar pre-operative and post-operative VHI-10 scores. Voice scores appear to be similar between the two groups in the pre-operative period, with only T2a patients showing significant improvement following surgery. Despite T2b patients having decreased vocal cord mobility, T2a disease often presents as an exophytic mass that prevents glottic closure. In this sense, voice outcomes between the two groups would be similar in the pre-operative period. However, T2a disease represents a superficial cancer, and resection allows the vocal cords to re-approximate appropriately. In the case of T2b, proposed to be a more advanced cancer, increased sacrifice of the vocal cord is often required, resulting in glottic incompetence. Although additional voice related measurements could be performed for objective examination of this phenomena, the clinical utility should be called into question, as VHI-10 is intended to be a subjective, patient oriented assessment of vocal characteristics [13]. Voice outcomes at 1 year were chosen as our group has previously demonstrated no significant change in voice quality after 1 year post-operatively (results not yet published).

Finally, organ preservation was substantially better with T2a disease, while total laryngectomy and organ sacrifice was seen in approximately one tenth of T2b patients. This rate of salvage laryngectomy is similar to Canis and colleagues, who reported a total laryngectomy rate of 17% in patients with T2b disease, although 7% of patients with T2a disease also required salvage laryngectomy in their study. While five-year laryngeal preservation rate appeared better for T2a disease, the low number of events may have contributed to a non-significant statistical result. Furthermore, some patients may die of unresectable disease and therefore artificially inflate the rate of laryngeal preservation, in either group.

There are limits to this study. Documentation of vocal cord mobility in T2 cancers was generally well recorded, however missing data did limit our population size slightly. As well, VHI-10 data was generally accessible, but was missing in approximately one third of patients. Both electronic and paper medical records were reviewed in order to increase data availability. Unfortunately, not all patients had voice outcome information documented adequately. Despite this, total available voice data was on par, or greater, than the majority of similar studies [14]. Voice outcome data is still lacking for T2 cancers in general, and further effort should be made to examine this group further. Lastly, this study was not designed to address radiotherapy outcomes compared to TLM outcomes in T2a vs T2b patients, but future investigations are warranted for this as well.

## Conclusion

T2 glottic cancer is a heterogeneous group that may be substratified into T2a and T2b on the basis of decreased vocal cord mobility. We have demonstrated worse voice outcomes in patients with T2b disease, as well as a significantly higher rate of salvage total laryngectomy. We also provide additional evidence to support decreased local control rates in patients with T2b disease. This study also demonstrates the possibility of repeated TLM procedures for recurrent disease in order to avoid radiotherapy. Further investigation should be aimed at determining the ideal treatment for patients with T2b disease in order to maximize both oncological and functional outcomes.

## Data Availability

Data sharing is applicable to this article as datasets were generated or analysed during the current study. Data sharing is unavailable for this study as it would compromise patient privacy. However, further information regarding the cases are available, within limits of patient privacy, upon request.

## Abbreviations

AJCC: American Joint Committee on Cancer
SCC: Squamous Cell Carcinoma
TLM: Transoral Laser Microsurgery
VHI: Voice Handicap Index

## References

[1] Jones T, De M, Foran B, Harrington K, Mortimore S (2016). Laryngeal cancer: United Kingdom National Multidisciplinary guidelines. J Laryngol Otol.

[2] Harwood AR, Deboer G (1980). Prognostic factors in T2 glottic cancer. Cancer.

[3] McCoul ED, Har-El G (2009). Meta-analysis of impaired vocal cord mobility as a prognostic factor in T2 glottic carcinoma. Arch Otolaryngol Head Neck Surg.

[4] Canis M, Martin A, Ihler F, Wolff HA, Kron M, Matthias C (2014). Transoral laser microsurgery in treatment of pT2 and pT3 glottic laryngeal squamous cell carcinoma—results of 391 patients. Head Neck.

[5] Spielmann P, Majumdar S, Morton R (2010). Quality of life and functional outcomes in the management of early glottic carcinoma: a systematic review of studies comparing radiotherapy and transoral laser microsurgery. Clin Otolaryngol.

[6] Mlynarek A, Kost K, Gesser R (2006). Radiotherapy versus surgery for early T1–T2 glottic carcinoma. J Otolaryngol..

[7] Jacobson BH, Johnson A, Grywalski C, Silbergleit A, Jacobson G, Benninger MS (1997). The voice handicap index (VHI): development and validation. Am J Speech Lang Pathol.

[8] Rosen CA, Lee AS, Osborne J, Zullo T, Murry T (2004). Development and validation of the voice handicap index-10. Laryngoscope.

[9] Kleinsasser O (1992). Revision of classification of laryngeal cancer, is it long overdue?:(proposals for an improved TN-classification). J Laryngol Otol.

[10] Martensson B, Fluur E, Jacobsson F (1967). Aspects on treatment of cancer of the larynx. Ann Otol Rhinol Laryngol.

[11] Trotti A, Zhang Q, Bentzen SM, Emami B, Hammond ME, Jones CU (2014). Randomized trial of hyperfractionation versus conventional fractionation in T2 squamous cell carcinoma of the vocal cord (RTOG 9512). Int J Radiat Oncol Biol Phys.

[12] Warner L, Lee K, Homer J (2017). Transoral laser microsurgery versus radiotherapy for T2 glottic squamous cell carcinoma: a systematic review of local control outcomes. Clin Otolaryngol.

[13] Batalla FN, Cueva MJC, González BS, Pendás JLL, Gil CG, Llames AL (2008). Voice quality after endoscopic laser surgery and radiotherapy for early glottic cancer: objective measurements emphasizing the voice handicap index. Eur Arch Otorhinolaryngol.

[14] Loughran S, Calder N, MacGregor F, Carding P, MacKenzie K (2005). Quality of life and voice following endoscopic resection or radiotherapy for early glottic cancer. Clin Otolaryngol.

